# Automating parasite egg detection: insights from the first AI-KFM challenge

**DOI:** 10.3389/frai.2024.1325219

**Published:** 2024-08-29

**Authors:** Salvatore Capuozzo, Stefano Marrone, Michela Gravina, Giuseppe Cringoli, Laura Rinaldi, Maria Paola Maurelli, Antonio Bosco, Giulia Orrù, Gian Luca Marcialis, Luca Ghiani, Stefano Bini, Alessia Saggese, Mario Vento, Carlo Sansone

**Affiliations:** ^1^Department of Electrical Engineering and Information Technology, University of Naples Federico II, Naples, Italy; ^2^Department of Veterinary Medicine and Animal Productions, University of Naples Federico II, Naples, Italy; ^3^Department of Electrical and Electronic Engineering, University of Cagliari, Cagliari, Italy; ^4^Department of Biomedical Sciences, University of Sassari, Sassari, Italy; ^5^Department of Information Engineering, Electrical Engineering and Applied Mathematics, University of Salerno, Salerno, Italy

**Keywords:** microscope, FLOTAC, semantic segmentation, object detection, veterinary, parasite eggs

## Abstract

In the field of veterinary medicine, the detection of parasite eggs in the fecal samples of livestock animals represents one of the most challenging tasks, since their spread and diffusion may lead to severe clinical disease. Nowadays, the scanning procedure is typically performed by physicians with professional microscopes and requires a significant amount of time, domain knowledge, and resources. The Kubic FLOTAC Microscope (KFM) is a compact, low-cost, portable digital microscope that can autonomously analyze fecal specimens for parasites and hosts in both field and laboratory settings. It has been shown to acquire images that are comparable to those obtained with traditional optical microscopes, and it can complete the scanning and imaging process in just a few minutes, freeing up the operator's time for other tasks. To promote research in this area, the first AI-KFM challenge was organized, which focused on the detection of gastrointestinal nematodes (GINs) in cattle using RGB images. The challenge aimed to provide a standardized experimental protocol with a large number of samples collected in a well-known environment and a set of scores for the approaches submitted by the competitors. This paper describes the process of generating and structuring the challenge dataset and the approaches submitted by the competitors, as well as the lessons learned throughout this journey.

## 1 Introduction

Grazing ruminants are exposed to gastrointestinal parasites, which can have very different implications on the host in terms of type and intensity of pathogenic effect and on the control practices required (Maurizio et al., 2023). Among all, gastrointestinal nematode infections are a common constraint in pasture-based herds and can cause a decrease in animal health, productivity, and farm profitability (Vande Velde et al., 2018). Knowledge about the presence and distribution of helminth infections is therefore crucial to plan effective parasite control programmes (Charlier et al., 2020). Thus, detecting, identifying, and quantifying the presence and spread of intestinal parasite infection is a crucial, but non-trivial task, usually performed with non-invasive tools, such as the microscopic examination of fecal samples, and, in particular, with techniques of fecal egg count (FEC). However, the manual count of parasite eggs requires relatively costly microscopes and a highly trained observer, who has to stay focused on the task for several hours, often resulting in count errors that lead to the prescription and use of inadequate dosage of drugs (Peña-Espinoza, 2018). Moreover, one of the most relevant problems regarding the standard approach for animal infection diagnosis is the apparatus (e.g., FLOTAC, Mini-FLOTAC, etc.) transport, which could take up to several hours from farm to laboratories, causing huge issues in terms of working time (Bosco et al., 2018). This is compounded if the samples obtained from farming reveal to be wrongly collected once analyzed with an optic microscope in a laboratory and, consequently, forcing a return to the farming for a new collection (Sabatini et al., 2023). As a consequence, these aspects have made clear the necessity of research and development of automatic systems for the localization, identification, and FEC. Artificial Intelligence (AI) based solutions have been gaining promising performance for these kinds of tasks, where a key role is played by Deep Learning (DL) approaches, such as Convolutional Neural Networks (CNNs) (Slusarewicz et al., 2016; Elghryani et al., 2020; Nagamori et al., 2020), thanks to their ability to learn the best data representation for the specific problem to be solved.

The Kubic FLOTAC Microscope (KFM) (Cringoli et al., 2021) represents a compact, low-cost, versatile, and portable digital microscope designed to autonomously analyze fecal specimens prepared with FLOTAC or Mini-FLOTAC (Cringoli et al., 2010, 2017), in both field and laboratory settings, for different parasites and hosts. Having been proven to acquire images comparable to the view provided by traditional optical microscopes, it is able to autonomously scan and acquire images from a FLOTAC or Mini-FLOTAC in a few minutes, allowing the operator to focus on a different task. Based on the KFM, the University of Naples Federico II organized the AI-KFM challenge, an international online competition hosted on Kaggle in which participants were asked to compete in fecal egg detection of gastrointestinal nematodes (GINs) in cattle, considering RGB images. The aim of the challenge was to support the research community by providing a standardized common experimental protocol, as well as a large number of samples collected in a well-known environment and a set of scores for competitors' approaches already available, so that a new solution for automatic FEC can be found and improved in a faster way. Each competitor could focus on different parts of the detector system pipeline in order to provide the best solution possible, for instance on the pre-processing step, in order to understand which could be the best transformation to enhance the researched features, or on the egg detector itself. In particular, the purpose is to advance the development of fully-automatic solutions for parasite eggs detection, providing a free-to-use and broad dataset, available from the beginning of 2022. This dataset comprises images derived from real-world samples, specifically cattle fecal samples processed using FLOTAC apparatuses, that include varying concentrations of eggs and diverse levels of contamination. While other datasets, such as Chula-ParasiteEgg-11 (Palasuwan et al., 2022), have been proposed in the literature, our competition dataset represents a unique case study. Unlike Chula-ParasiteEgg-11, which features images deliberately focused on individual eggs by operators, our dataset allows for analysis directly in the field without requiring a laboratory setting or operator intervention. This characteristic ensures a more realistic representation of what an automatic egg detector encounters during a scan session, where images may contain hundreds of eggs or none at all. Furthermore, the Chula-ParasiteEgg-11 dataset includes eggs captured under varying conditions, using different apparatuses, light settings, and focus points. In contrast, our dataset for the AI-KFM challenge is specifically designed to train models optimized for the Kubic FLOTAC Microscope, thereby leveraging all the advantages of this tool.

The provided dataset for the challenge, combined with the selection of reliable devices from the FLOTAC family, the capability to conduct on-field scan sessions, and the adoption of state-of-the-art deep learning models, has enabled us to develop an optimized system for the Kubic FLOTAC Microscope. This system significantly simplifies the work of researchers and veterinarians, surpassing the efficiency of current state-of-the-art solutions. Additionally, to establish the competition as a standardized experimental platform, we continue to welcome new solutions. This ongoing acceptance fosters collaboration within the research community and offers a real-world scenario for testing new methodologies.

In this paper, we provide a comprehensive overview of the first AI-KFM challenge, focusing on the various aspects that were covered during the competition. We describe in detail the process of generating and structuring the dataset, also providing insights into the challenges and limitations that we faced during this process and how we overcame them. Moreover, we describe the different approaches that were submitted by the participants, detailing the methodologies and techniques that were employed. Finally, we reflect on the lessons learned throughout this journey, highlighting the key takeaways from the competition and the implications for future research in this field. We discuss the importance of collaboration and standardization in promoting advancements in object detection in veterinary medicine, and how the AI-KFM challenge has contributed to this effort. To sum up, this paper provides a comprehensive overview of the AI-KFM challenge and its significance for advancing the field of object detection in veterinary medicine.

The structure of the paper is organized as follows: Section 2 describes the existing solutions in the literature; Section 3 details the AI-KFM challenge; Section 4 summarizes the methodologies implemented by the participants; Section 5 shows the obtained results; Section 6 will contain a brief discussion of the results obtained with their implications, finally Section 7 provides some conclusions.

## 2 Related works

Several manual and automatic FEC techniques already exist. Table 1 summarizes the limitations of the most used ones, highlighting the need for more reliable approaches. Indeed, while solutions such as the Parasight System and VETSCAN IMAGYST have shown better performance than most competitors (Scare et al., 2017; Cain et al., 2020; Nagamori et al., 2020, 2021), they are only validated on a restricted range of hosts and are therefore limited in their applicability. On the other hand, solutions such as the Lab-on-disk Platform and DAPI offer high-quality images and good identification accuracy, but their high cost and lack of portability make them unsuitable for on-field use, which prevents the scanning process from being started directly after sample collection, thereby increasing analysis times (Sukas et al., 2019; Inácio et al., 2021).

**Table 1:** List of the most used existing solutions for manual and automatic fecal egg count, reporting for each a brief description, possible hosts and parasites, strengths and the main limitations.

**System**	**Principle**	**Hosts**	**Parasites**	**Advantages**	**Limitations**
FECPAKG2 (Tyson et al., 2020)	High-throughput technological system for on-field sample processing	Ruminants, humans	GINs, soil-transmitted helminths (STH)	Automated detection and count, remote parasite detection and data online management	Low sensitivity and accuracy
Parasight system (Slusarewicz et al., 2016)	Based on a fluorescent egg staining and a smartphone to capture images	Horses	Strongyles, parascaris equorum	2.5 min test time, less variables and more accurate than McMaster technique	Validated only on horses
Lab-on-disk platform (Sukas et al., 2019)	Based on a combined gravitational and centrifugal rotation	Humans, pigs	STH, schistosoma mansoni, ascaris suum	High quality of images, permitting a good identification and count	High cost, limited use on field
Automated robotic system (Lu et al., 2018)	Based on an automated X-Y stage, autofocus and scan provided by LabVIEW GUI	Monkey, dogs, cattle, sheep	*Trichuris* spp, *Toxocara* spp, *Strongyles, Isospora* spp, *Eimeria* spp	Inexpensive, compact, possibility to use
fluorescence	Compatible with McMaster only, not validated
Automated Diagnosis of Intestinal Parasites (DAPI) (Inácio et al., 2020)	Based on a motorized system and using a digital camera and machine learning software	Dogs	*Ancylostoma* spp, *Toxocara* spp, *Trichuris* spp, *Giardia* spp	Automated detection of eggs through machine learning software	High cost, not portable, not validated
Telenostic System (Elghryani et al., 2020)	Automated digital microscope with a 10 × x lense using machine learning software	Cattle	GINs	High level of agreement between the prototype and manual systems of FEC	Validated only on cattle, 40 min analysis
VETSCAN IMAGYST (Nagamori et al., 2020)	Composed of a digital slide scanner and machine learning software	Dogs, cats	Ancylostomidae, *Toxocara* spp, *Trichuris* spp, Taeniidae	The system allows detection and count of eggs within 15 min	High cost, not portable, validated on dogs/cats only

As reported in several comparative papers (Bosco et al., 2014; Godber et al., 2015; de Castro et al., 2017; Cools et al., 2019) the FLOTAC and Mini-FLOTAC systems outperform different competitors, such as McMaster, Wisconsin, Kato-Katz and FECPAK in FEC accuracy and sensitivity.

In literature, several works cover the problem of detecting automatically parasite eggs in samples for FEC and introduce the corresponding solutions, each one with different approaches and techniques.

Most studies rely on private datasets independently extracted by research groups (Naing et al., 2022; Kumar et al., 2023a; Suwannaphong et al., 2023), utilizing various state-of-the-art Convolutional Neural Networks (CNNs) such as AlexNet (Krizhevsky et al., 2012), ResNet (He et al., 2016), and YOLO (Redmon et al., 2016). Notably, the work by Mayo et al. (2022) is unique in adopting a workflow that integrates a Generative Adversarial Network (GAN) alongside a standard object detector (Faster R-CNN). Additionally, several solutions introduced after the conclusion of the AI-KFM Challenge employ the Chula-ParasiteEgg-11 dataset from Palasuwan et al. (2022), which includes 11 classes of parasite eggs. Studies such as those by Pedraza et al. (2022), Ruiz-Santaquiteria et al. (2022), Wang et al. (2022), AlDahoul et al. (2023), and Rajasekar et al. (2023) utilize this dataset with a broad range of deep learning models, primarily leveraging CNNs.

Moreover, given the advancements provided by data processing algorithms in conjunction with the models themselves, incorporating such algorithms into the data elaboration workflow should be considered essential.

This integration is a complex and time-consuming task. Various automatic or semi-automatic solutions have been developed, but each has limitations that hinder widespread adoption. These limitations range from apparatus quality to the capability of executing processes immediately after sample collection. Most automatic systems require significant computational resources, leading to solutions that necessitate specialized hardware designed for laboratory use. Consequently, veterinary researchers are often required to transport samples from animal farms to laboratories and wait for extended periods before analysis can commence, increasing the risk of data degradation during transit.

In order to remedy these limitations, the University of Naples Federico II provides the KFM, an extremely portable and user-friendly microscope designed to be adopted by any kind of operator for on-field analyses, in order to retrieve a dataset which can be adopted for automatic models for parasite eggs detection, which can be later integrated in the KFM ecosystem, making it the ideal solution for the task described so far.

## 3 AI-KFM 2022 challenge

Given these premises, the University of Naples Federico II proposed the AI-KFM 2022 Challenge^1^ whose aim was to make competitors build an algorithm for the automatic detection and count of parasite eggs. In particular, during the challenge, competitors had to design and develop a system composed of a pre-processing pipeline for a previously collected dataset, and a model that can be used with a dynamic test set and for different tasks since the purpose was to define an architecture protocol and to obtain robust models. The solution had to be submitted on the dedicated Kaggle page, where also inferences results are still available.

However, before understanding how the KFM itself and the challenge dataset are composed, it is needed to focus on apparatuses adopted to extract it, which are the ones the KFM was built on: the FLOTAC and the Mini-FLOTAC.

### 3.1 FLOTAC and Mini-FLOTAC

**Figure 1:**
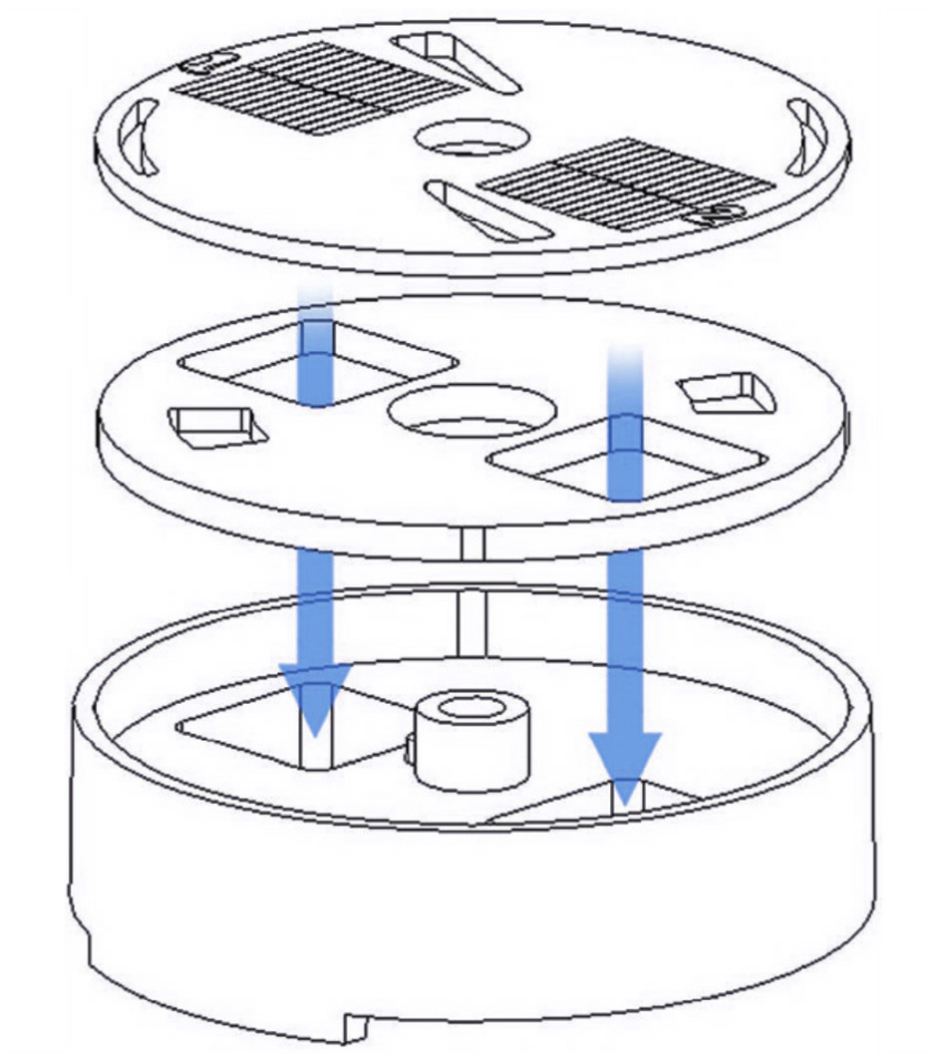
Physical components of the FLOTAC apparatus. The core device is composed of a reading disc, a translation disc and a base. Arrows indicate the flotation chambers.

The FLOTAC family apparatuses, which are FLOTAC, Mini-FLOTAC and Fill-FLOTAC, constitute the heart of the system, representing fecal sample collectors. The FLOTAC apparatus consists of a cylindrical device made of polycarbonate amorphous thermoplastic, chosen for its excellent light transmission, robustness to washing and re-use, resistance to high heat, and high dimensional stability. Figure 1 illustrates the physical components of the device, which include the base, translation disc, and reading disc. The FLOTAC contains two 5-ml flotation chambers that are specifically designed for optimal and accurate examination of large fecal sample suspensions.

**Figure 2:**
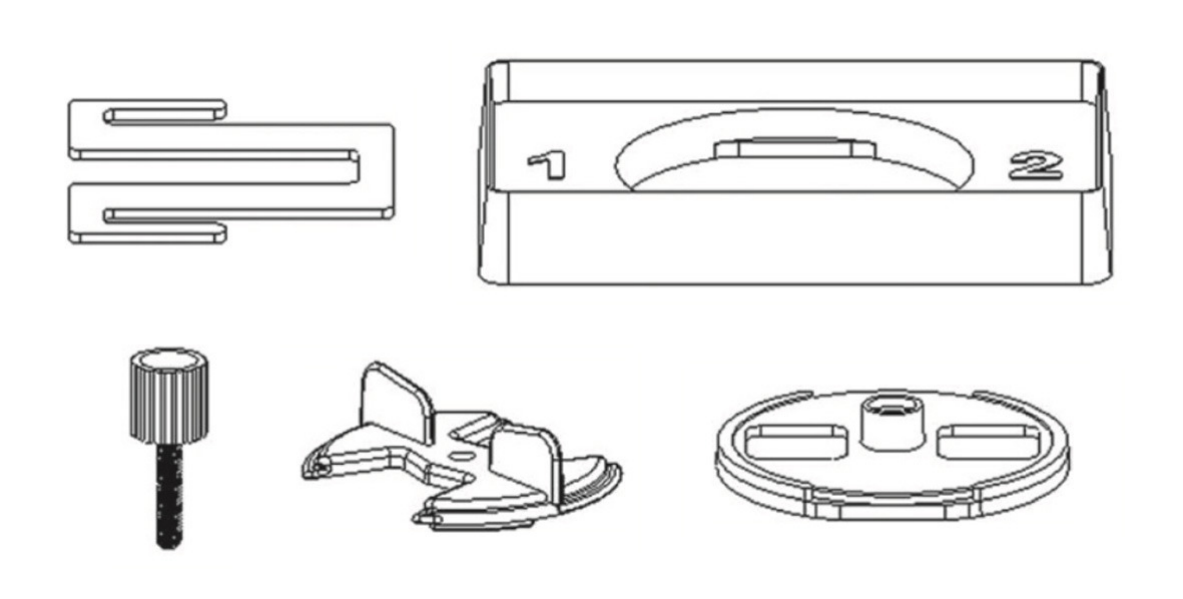
Accessories of the FLOTAC apparatus. These include microscope adapter, centrifuge adapter, screw, key and bottom.

In addition to the FLOTAC device itself, several accessories are also provided to ensure proper functioning of the apparatus during centrifugation and examination with microscope, as depicted in Figure 2. These accessories include the microscope adapter, centrifuge adapter, screw, key and bottom.

**Figure 3:**
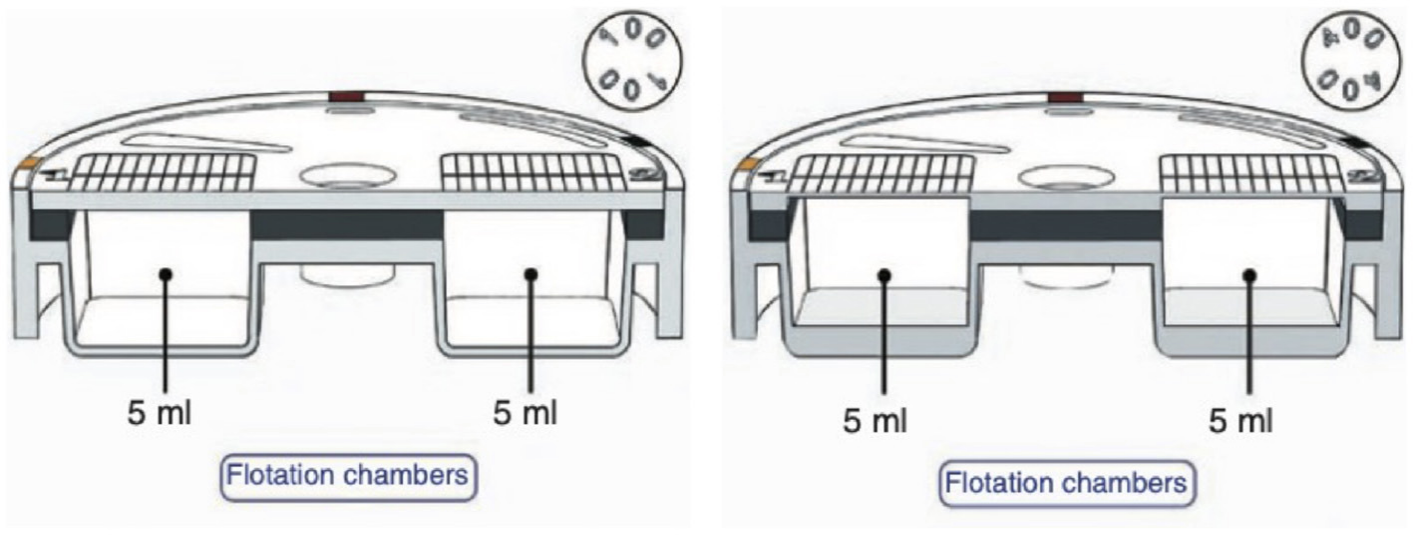
Structure of FLOTAC-100 apparatus and of FLOTAC-400 apparatus.

There are two versions of the FLOTAC: the FLOTAC-100, which allows a maximum magnification of 100x, and the FLOTAC-400, which allows a maximum magnification of 400x. FLOTAC-400 is an enhanced version of the FLOTAC-100, since it allows microscopic diagnosis at greater magnification, a crucial aspect for the detection of intestinal protozoa. The FLOTAC-100 version, however, is still suggested for both helminth eggs and larvae diagnosis and teaching purposes, as the reading disc is thicker and more robust than the FLOTAC-400 one and the flotation chambers can be filled more easily. Structural differences between the two apparatuses can be seen in Figure 3.

**Figure 4:**
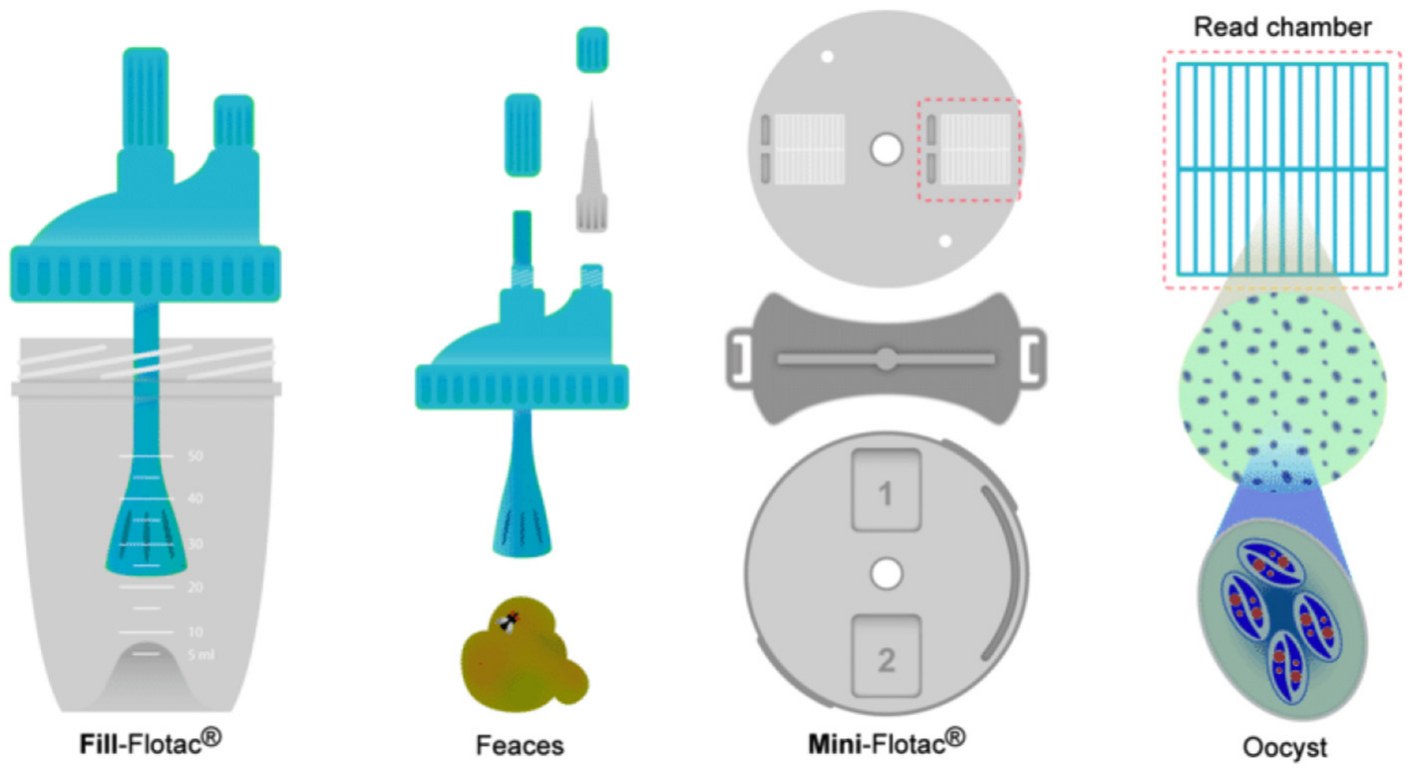
Fill-FLOTAC components, Mini-FLOTAC components and Mini-FLOTAC key.

As the name suggests, the Mini-FLOTAC is an evolution of the FLOTAC apparatus, from which it differs in its slimmer shape and the absence of the translation disc. This new composition makes the Mini-FLOTAC easier to carry and assemble while, given its size, the total flotation chamber capacity is only 2 ml, 1 ml for each chamber, with a maximum allowed magnification of 400 × . Besides the key which enables the Mini-FLOTAC assembly, the Mini-FLOTAC kit includes also a device called Fill-FLOTAC, which is a disposable sampling device composed of a container, a collector, and a filter. Its components, along with the Mini-FLOTAC apparatus and its key, are shown in Figure 4.

**Figure 5:**
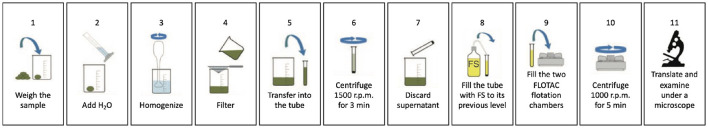
The 11 operating steps of the FLOTAC device.

It is important to note that the quality of data collected is heavily reliant on the correct preparation of the FLOTAC devices, which involves a protocol consisting of 11 steps (depicted in Figure 5). The process begins with a tube of final sample obtained by filtering and centrifuging a homogeneous solution of water and fecal sample, removing the supernatant, and mixing the remaining sediment with a flotation solution. The resulting solution is then poured uniformly into the two 5 ml flotation chambers of the FLOTAC. The apparatus is then sealed and subjected to centrifugation, causing the parasite eggs shields to float and become easily analyzable.

**Figure 6:**
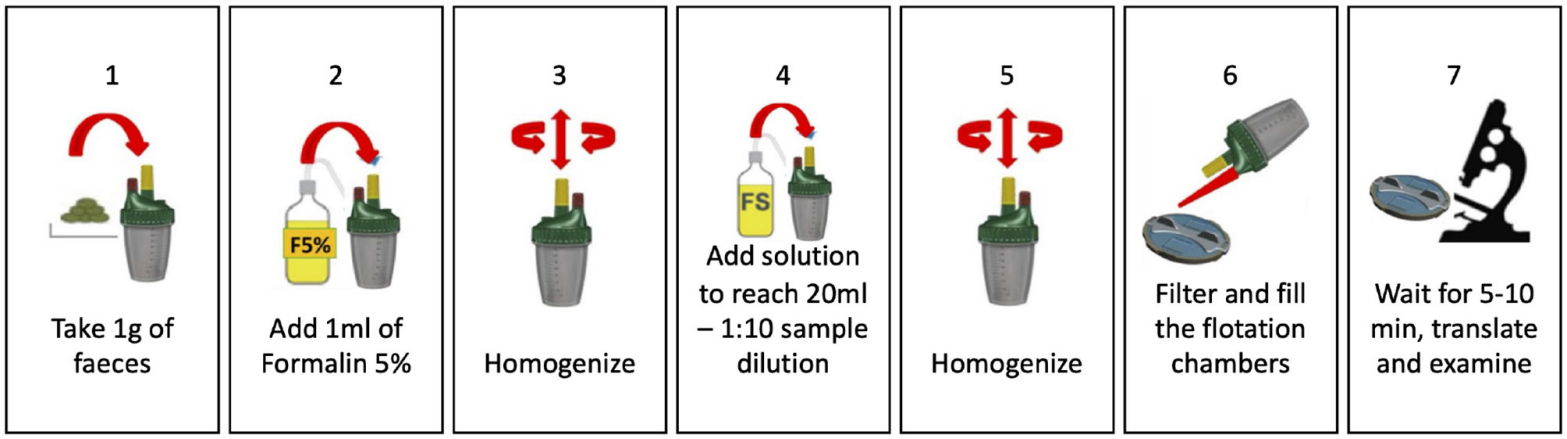
The seven operating steps of the Mini-FLOTAC device.

The FLOTAC technique has the drawback of requiring a centrifuge, which limits the KFM's ability to function on-site. However, this problem can be overcome by using the Mini-FLOTAC, which does not require centrifugation, as reported in Barda et al. (2013). By using the Fill-FLOTAC that comes with the Mini-FLOTAC, some standard FLOTAC steps can be replaced. Figure 6 shows the operating steps of the Mini-FLOTAC, which only require seven steps and do not require a centrifuge.

### 3.2 The microscope structure

Performing an analysis of FLOTAC and Mini-FLOTAC apparatuses by a human operator with an optical microscope can be a time-consuming and arduous process. Furthermore, due to the distance between farms and laboratories, any errors in the application of the FLOTAC technique may only be discovered during the analysis in the laboratory, requiring the operator to discard the sample and return to the farm to collect another one. Therefore, it has become imperative to develop an automatic and portable tool for fecal egg count that can be easily used on-site to save time and energy. To meet these requirements, the Kubic FLOTAC Microscope, or KFM, was designed and constructed.

**Figure 7:**
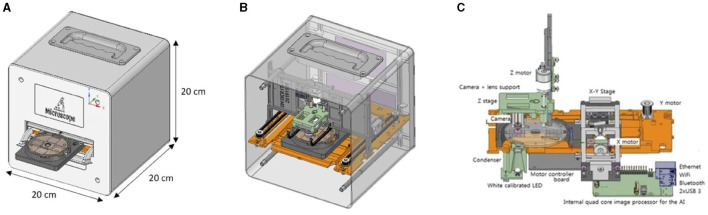
FreeCAD and Design Spark Mechanical CADs of the KFM **(A)** external view and **(B)** internal view and **(C)** schematic diagram showing mechanical, electronic and optical systems of the KFM.

The KFM is made up of a combination of electro-mechanical components that allow for both manual and automatic scans of the FLOTAC/Mini-FLOTAC reading discs. The firmware of the KFM enables three-axial camera movements, remote interactions, and retrieval of scans. External agents such as the KFM web interface and a smartphone application can be used to connect to the KFM hardware, and the KFM AI server can process scans for parasite egg detection. The FLOTAC/Mini-FLOTAC apparatus is placed on the KFM slide-out tray and then inserted into the device. The tray is then withdrawn inside, and 3D landmarks are automatically set on the upper-left corners of the two flotation chambers. This ensures that the scan process starts from a standard and well-known position. Essentially, the KFM is an XYZ-motorized stage for microscopy. The KFM 3D mechanical design was created using FreeCAD and Design Spark Mechanical, and a diagram of the design is shown in Figure 7.

The optical component of the KFM includes an LED light with a condenser for brightness adjustments and a digital camera that allows for adjustable magnifications of 100 × , 200 × , and 300 × , with a maximum resolution of 8 MPixels (3,264 × 2,448 pixels) and an image size of 1,024 × 768 (0.8 MP). The three-axis positioning system of the motorized stage is unique, utilizing open-loop stepper motors and precision translation stages to achieve precise motion control in three dimensions.

### 3.3 Dataset

The dataset consists of images acquired by the KFM, showing example of GINs. Each FLOTAC/Mini-FLOTAC sample generates very high-resolution images, split in hundreds of overlapping patches, and saved in .jpeg or .png format, resulting in thousands of images made available for the competition, unpublished and not provided to other people before. Each patch may or may not contain parasites' eggs. For training data, participants are also provided with region of interest (ROI) files containing, for each image, the coordinates of the bounding box of parasites within it, as identified and segmented by an expert operator. Test data is provided without the ROI files. Training and test datasets belong to different acquisition samples (i.e. different FLOTAC and/or Mini-FLOTAC devices).

The dataset consists of images coming from 11 different FLOTAC/Mini-FLOTAC, of which seven have been used for the Training set and four for the Test set. Each acquisition in the Training set has two folders:

- Images: containing the images belonging to the same acquisition. Images in this folder can be considered as patches (partially overlapping) extracted from a bigger image;
- Masks: containing the coordinates of the boxes surrounding the eggs, saved as .roi files. Each .roi file, reports the four coordinates of the box as four-elements vector *X*_*min*_, *Y*_*min*_, *X*_*max*_, *Y*_*max*_.

The whole dataset is currently available in the Data section of the Kaggle competition page.^2^

The challenge allowed competitors to make their models inferences on different test sets, which were available in different periods of time. In particular, the test happened at these two different moments:

- During the first period of the competition (day 1 up to 2 day to the deadline), a test sample (images coming from a single FLOTAC and/or Mini-FLOTAC device) is available for participants to test the performance of their models (Test 1);
- On the last day, a new test dataset (with images coming from more than one FLOTAC and/or Mini-FLOTAC device) is released (Test 2).

The aim of dividing the test set into two different moments is to test the generalization ability of the submitted models. This is very important as, despite KFM having an extremely low intra-operator variability, the preparation of the sample can affect the acquired image quality. Both these datasets are available on the Data section of the AI-KFM 2022 Challenge Kaggle page.

A subset of the test data has been used for the public leaderboard (i.e., a leaderboard that is updated in real-time as soon as a participant submits a prediction file), while the remaining part will be only used for the private leaderboard (that is made available at the competition end). During the first period, 100% of the data have been used for the public leaderboard, while during the second period (the last day), only 5% of the data has been used for the public leaderboard. Teams will be allowed a maximum of 1 submission per day. This means that they will have a single submission on the last day to submit a prediction for the real test set.

### 3.4 Tracks

The available tracks for this competition were two, where the first was mandatory and the second was optional. In particular, the first one (Track 1) consists of the detection of parasites' eggs, while the second track evaluates the inference time on the test set. For both tracks, the participants were asked to submit a Unix executable or a Python code. The submission would be used to reproduce the obtained results and to measure inference speed under the same workload conditions in Track 2.

### 3.5 Performance evaluation

The performance of the competition for eggs detection (Track 1) is evaluated on the F1-Score at different intersection over union (IoU) thresholds. The IoU of a proposed set of object pixels and of a set of true object pixels is calculated as in Equation 1:


(1) $$\begin{array}{l} IoU\left(A,B\right)=\frac{A\cap B}{A\cup B} \end{array}$$

The metric sweeps over a range of IoU thresholds, at each point calculating an F1-Score. The threshold values range from 0.5 to 0.95 with a step size of 0.05: (0.5, 0.55, 0.6, 0.65, 0.7, 0.75, 0.8, 0.85, 0.9, 0.95). In other words, at a threshold of 0.5, a predicted object is considered a “hit” if its intersection over union with a ground truth object is >0.5. At each threshold value *t*, the F1-Score value is calculated based on the number of true positives (TP), false negatives (FN), and false positives (FP) resulting from comparing the predicted object to all ground truth objects as in Equation 2:


(2) $$\begin{array}{l} F_{1}\left(t\right)=\frac{2TP}{2TP+FN+FP} \end{array}$$

A true positive is counted when a single predicted object matches a ground truth object with an IoU above the threshold. A false positive indicates a predicted object had no associated ground truth object. A false negative indicates a ground truth object had no associated predicted object. The average F1-Score ($F_{1}^{avg}$) of a single image is then calculated as the mean of the above F1-Score values at each IoU threshold, as shown in the Equation 3:


(3) $$\begin{array}{l} F_{1}^{avg}=\frac{1}{\left|thresholds\right|}\underset{t}{\sum }F_{1}\left(t\right) \end{array}$$

It is worth pointing out that we used a standard performance metric for object detection tasks in biomedical domain (Hicks et al., 2022; Rainio et al., 2024), especially for the detection of parasite eggs, where both precision and recall are crucial factors. Indeed, the F1-Score is their harmonic average. This makes the metric widely used in different works proposed in the literature (Kumar et al., 2023b), focusing on the same tasks.

Lastly, the score returned by the competition metric is the mean taken over the individual average F1-Scores of each image in the test dataset.

The performance of the competition for Track 2 is computed in terms of minutes to process a single acquisition, consisting of several patches, according to the following formula in Equation 4:


(4) $$\begin{array}{l} score=2*\frac{F_{1}*T_{n}}{F_{1}+T_{n}} \end{array}$$

where *T*_*n*_ is the min–max normalized execution time (median value over 10 repetitions) between all the participants.

## 4 Proposed methods

Obtaining a large dataset of images containing parasite eggs shields is quite time-consuming but easy and straightforward, since there are several steps which may take some time but are either common to the standard analysis process, like the FLOTAC/Mini-FLOTAC preparation, or almost completely autonomous, like the KFM scan.

A dataset obtained with this procedure is the one adopted by AI-KFM 2022 Challenge competitors in order to design and develop a robust and efficient processing, training and inference pipeline. Some of these solutions will be detailed in this section.

In particular, a total of three teams participated in this first-year competition, out of which: (i) only two successfully submitted the prediction for both test-set moments and (ii) only one took part to both tracks.

The participants proposed AI-based solutions, exploiting CNNs for the automatic extraction of the features related to the task to be solved. The first methodology is proposed by the PAttern Recognition Applied SardInia TEam (PARASITE), coming from the University of Cagliari and the University of Sassari, and it consists of a system composed of a Mask R-CNN and a post-processing filter for false positives removal. The second approach has been implemented by MIVIA Lab, from the University of Salerno and it consists of a system composed of a U-Net and a post-processing filter for bounding boxes extraction. Unfortunately, the members of the third team, that is Saksham_Aggarwal, did not provide the details about their solution, taking part in the competition without preparing the submission for Test 2. As a consequence, we will only describe the methodology implemented by PARASITE and MIVIA Lab.

### 4.1 PARASITE submission

The problem of automatic detection and the consequent count of gastrointestinal parasite eggs through microscopic examination of fecal samples can be addressed as a segmentation problem. The solution submitted by the PARASITE team of the University of Cagliari and the University of Sassari is based on the fine-tuning of famous CNN proposed in the field of object segmentation and detection for medical imaging.

#### 4.1.1 Adopted architecture: Mask R-CNN

In particular, the submitted solution is based on Mask R-CNN (He et al., 2017), a CNN-based framework that allows instance segmentation, which is object detection followed by semantic segmentation that allows understanding of which pixels belong to the object. In this work, it was used the Python implementation that is freely downloadable from Abdulla (2017).

**Figure 8:**
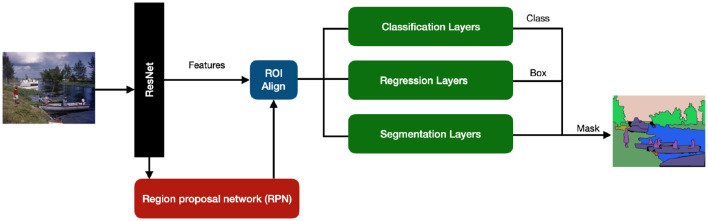
The PARASITE solution is based on an R-CNN Mask architecture. Mask R-CNN produces three outputs: a class label, a bounding-box offset, and an object mask for each candidate item.

**Figure 9:**
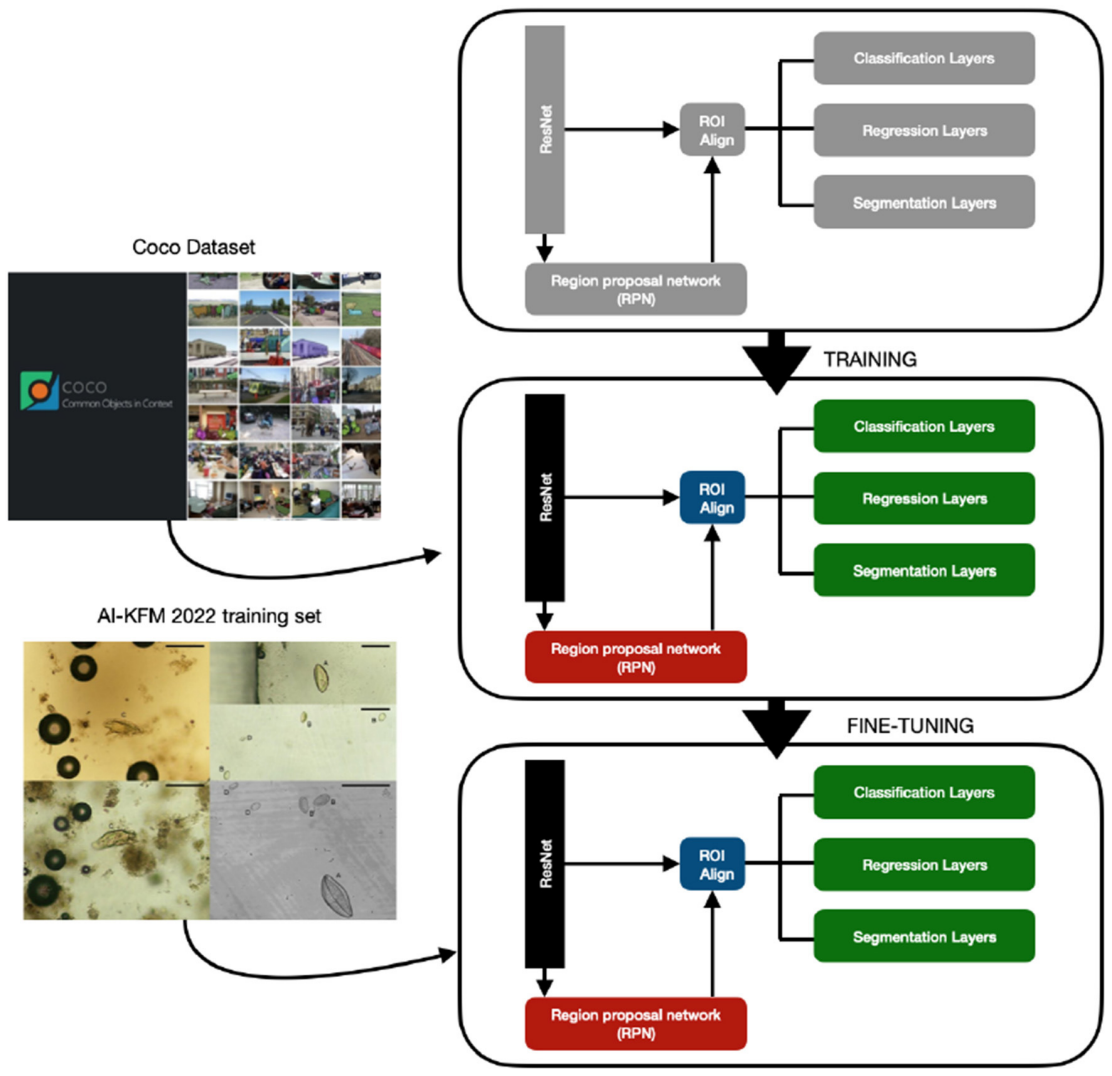
Fine-tuning of on a Mask R-CNN pre-trained model using the AI-KFM 2022 training set.

Mask Region-based CNN (R-CNN) is an extension of Faster R-CNN (Ren et al., 2017) and it adopts a two-stage procedure, as shown in Figure 8. To estimate the position of bounding boxes, the first stage uses a fully convolutional network called Region Proposal Network (RPN) followed by a RoI align layer to detect multiple, scaled or overlapped objects in an image. In the second stage, the RoI misalignments in the RoIAlign layer are corrected and then a classification, a bounding box regression, and the extraction of a binary mask are performed in parallel. The bounding boxes and the segmentation masks of the objects present in an image are detected at the pixel level and a class label is assigned to them. The proposed solution is based on a pre-trained model on the MS COCO dataset; the fine-tuning was done on the AI-KFM 2022 training set (Figure 9).

In particular, the selected model for the submission is trained on a subset of the available training set (80%) and validated on the remaining subset (20%) for 100 epochs with a learning rate of 10^−3^ and early stopping after 20 epochs in case of improvements lack.

Data augmentation includes random horizontal and vertical flips, translation operation of maximum 20 pixels and crops up to 0.2% of the whole image.

#### 4.1.2 Post-processing

**Figure 10:**
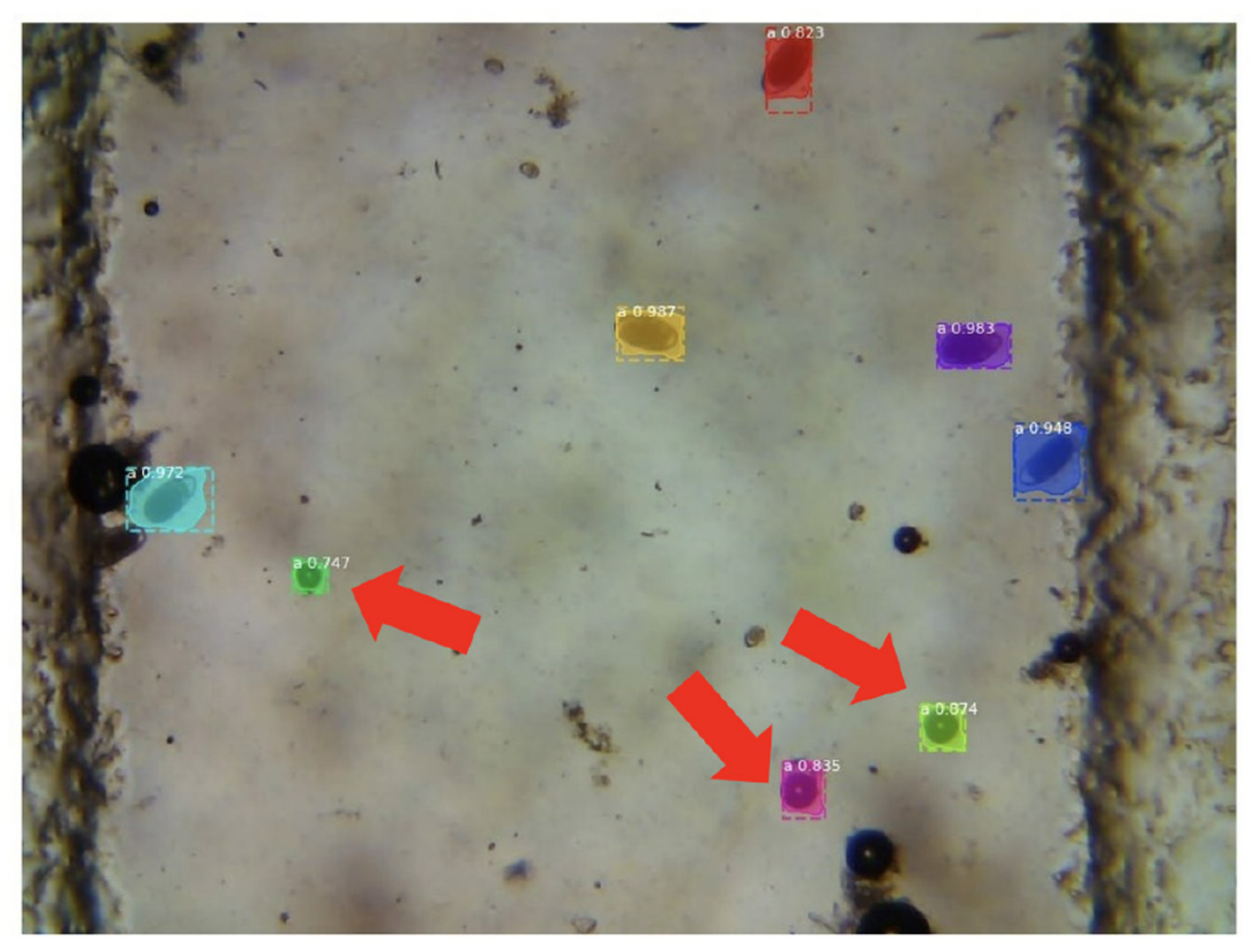
Output of the fine-tuned R-CNN Mask model. The system produces false positives in the presence of air bubbles.

Since the neural network incorrectly classifies air bubbles and other details as parasites, resulting in many false positives (Figure 10), the outputs obtained from the network are post-processed to reduce false positives. In particular, since the parasites have a double edge, while the air bubbles do not have these characteristics, a threshold on the number of pixels that constitute the edges of the object can be significant in discriminating true positives and false positives.

**Figure 11:**
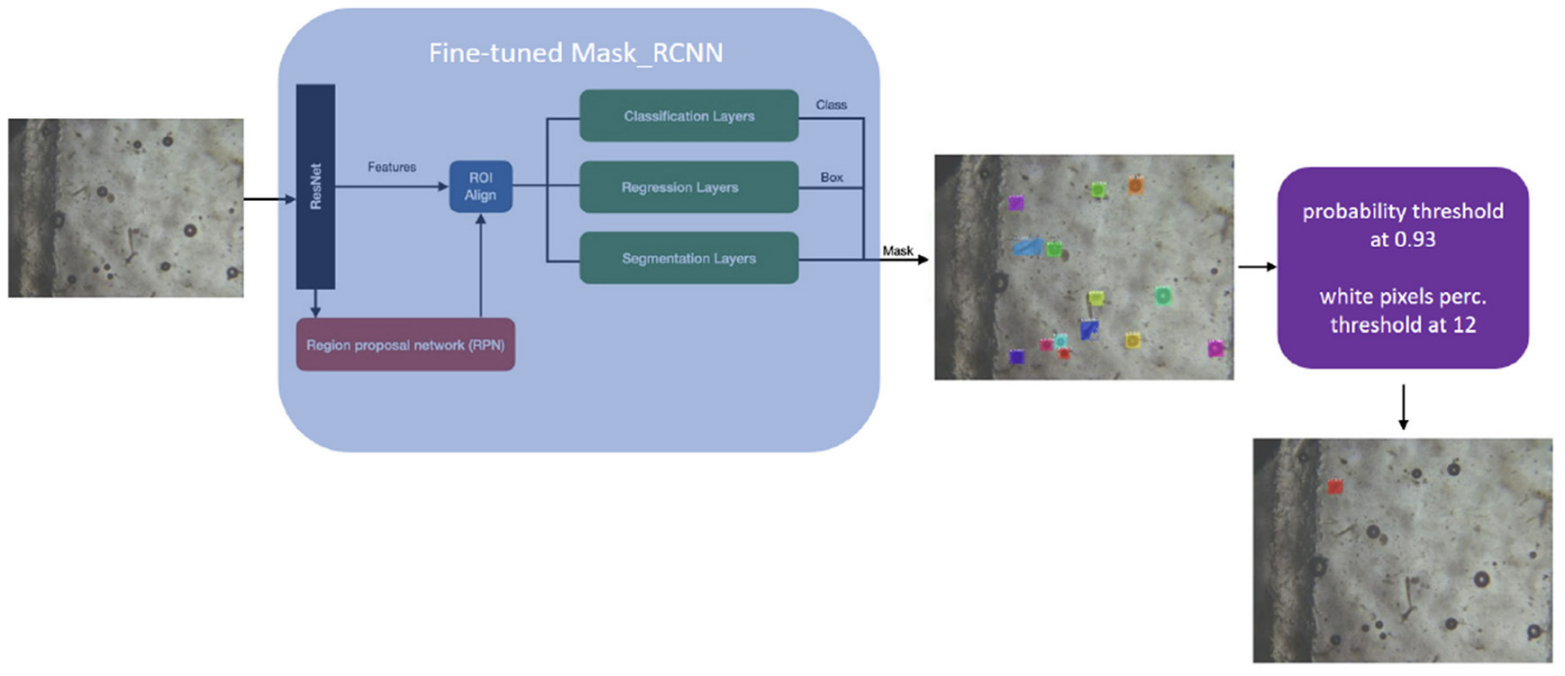
Complete method of the PARASITE team: the system includes a post-processing phase based on edge detection to avoid false positives.

Each identified item with a chance of being parasites higher than 93% is then post-processed using a Canny edge detector (lower threshold: 10, upper threshold: 50), and samples with a proportion of white pixels larger than 12% have been selected. The final scheme of the solution proposed by the PARASITE team is shown in Figure 11.

The model obtained with this architecture has been compared to a fine-tuned YOLOv5 model, which had similar performances but did not benefit from the post-processing step, thanks to which the original model was capable of filtering false positives.

### 4.2 MIVIA Lab submission

The solution proposed by MIVIA Lab, according to the team argument about its design and proposal, was conditioned by the limited amount of data provided, since it is not based on a typical Single-Shot Detector (SSD) architecture, but on a U-Net, since it is one of the most adopted architectures for Medical Imaging tasks with little data available (Liu et al., 2020). Although U-Net is born for segmentation, it has been also used for the detection (Dai et al., 2015; Rajchl et al., 2016). A naive approach could be to create the semantic mask to use for training the network directly from the bounding boxes. Anyway, it would prevent the network from focusing only on the regions of interest (corresponding to the pixels of the object).

Given the limited amount of data to train the system, and inspired by Dai et al. (2015), the proposed solution is a U-Net trained by using segmentation masks automatically generated starting from bounding boxes by exploiting the morphological properties of objects to be detected.

**Figure 12:**
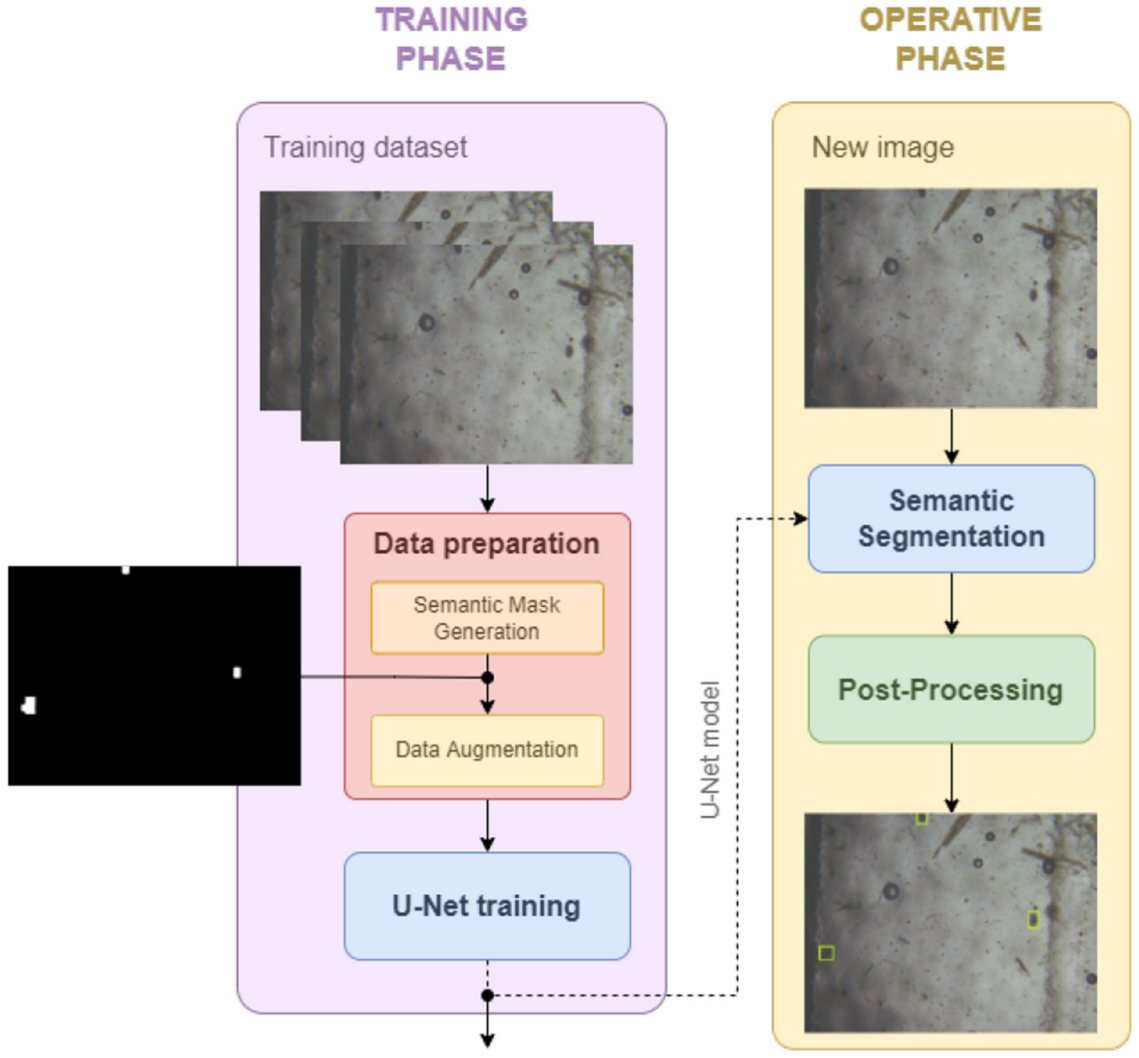
Training and operative phases. *Data preparation*, to get the segmentation mask from the bounding box and augment the data. *U-Net Training*, to obtain the model to be used at the operating phase in order to compute the eggs segmentation mask. *Post-processing*, to return the bounding boxes given the segmentation mask.

The architecture of the proposed system is shown in Figure 12. At training phase, the segmentation masks are automatically generated and then augmented 4.2.1 before training U-Net 4.2.2. At operating phase, the trained network is employed and a connected component analysis is performed to obtain the bounding boxes.

#### 4.2.1 Data preparation

Given the bounding boxes, the segmentation masks should be extracted in order to feed the network during the training phase. Even if a wide literature exists (Rajchl et al., 2016; Gröger et al., 2022), including thresholding, region-based, clustering, and NN, a common approach is pixel clustering (Zaitoun and Aqel, 2015). Indeed, it groups pixels based on the object shape, thus it is thought for situations in which objects have well-defined shapes. Furthermore, in order to deal with the fact that clustering may not guarantee the creation of continuous areas, mathematical morphology operators were adopted (Dougherty, 2018).

**Figure 13:**
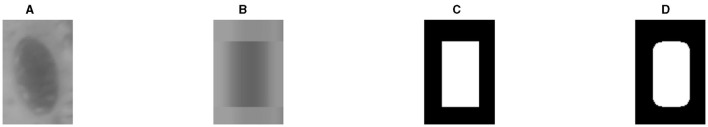
The generation of the segmentation mask for each cell, starting from the patch included in the bounding boxes, converted in grayscale **(A)**, is based on the following steps: **(B)** k-means clustering, **(C)** thresholding, **(D)** mathematical morphology.

In more detail, the K-means clustering algorithm has been adopted. Through an iterative process, starting from the original bounding box (Figure 13A), it groups the image pixels into K classes based on their attributes (texture, intensity, etc.). The goal is the minimization of the total intra-group variance. In the end, the centroid or midpoint of each group and the distance map are released by the algorithm (Figure 13B). The final result is obtained by running the algorithm 50 times with different random initialization, setting ϵ = 1.0 and a maximum number of iterations to 10. Subsequently, thresholding (binary + Otsu) is applied on the distance map to obtain a segmentation mask (Figure 13C). The patches are further processed by applying mathematical morphology operations to refine the images (Figure 13D): (i) *opening*, to remove spurious objects resulting in false positives, (ii) *closing*, to make the regions continuous. The size of the structuring element is determined based on an analysis of the bounding box sizes, which reported a minimum dimension of 40 × 40 pixels. Consequently, a circular structuring element with a diameter of 19 pixels is used.

In order to increase the size of the dataset, standard data augmentation techniques have been performed. Inspired by Suwannaphong et al. (2021), vertical and horizontal flippings were adopted. Indeed, rotation transformation has been discarded since many cells are on the edge of the images, and applying rotation and cropping would have eliminated such samples while using padding would have created artifacts. For the same reason, also the shifting operation is discarded.

#### 4.2.2 Adopted architecture: U-Net

Each image is resized before feeding the network with zero-order spline interpolation. A vanilla version of U-Net with a resolution of 572 × 572, has been used. U-Net is an encoder-decoder architecture, that introduces the skip connections between the contracting section (encoder) and the expansion section (decoder) so as to recover the spatial information lost due to pooling levels. They allow to maintain a high resolution of the output without increasing the complexity of the network and, therefore, have the main advantage to perform well even in presence of a small amount of training data (Liu et al., 2020).

For the training, the adopted cost function is boundary loss. The main advantage behind this choice is that it allows for mitigating the difficulties encountered during training caused by the high imbalance between foreground and background pixels. Indeed, boundary loss uses a distance metric based on contours and not on regions (Kervadec et al., 2019). The chosen optimizer is Ranger (Wright, 2019). It combines Rectified Adam (RAdam) with LookAhead optimizer to work faster and more stable on loss surface. The optimization algorithm was run for 1,000 epochs with a linear learning rate {10^−2^, 10^−5^}.

#### 4.2.3 Post-processing

During the operating phase, the mask obtained from the U-Net is used to extract the bounding boxes of the connected components, using 8-connectivity, which is a standard approach in medical imaging (Salvi et al., 2021).

## 5 Results

In this section, results regarding both the KFM hardware and the competitors solutions performances will be detailed and evaluated. Both these metrics should be considered in order to obtain an estimation of the quality and the work time the KFM system, composed of the scan process and the inference process, has.

In this evaluation, the accuracy of the FLOTAC itself is not taken into account, given the already proven accuracy and reliability of the apparatus, reported in the Related work section, and given the fact that the KFM system itself has been built upon the FLOTAC apparatus, therefore considering other apparatuses is not useful for the KFM system evaluation.

### 5.1 KFM hardware performances

When the KFM hardware has been tested for the first time, given the limitations of the first components adopted and the not-optimized version of the firmware, the KFM scan process resulted not so promising. Indeed, from the device loading to the generation of the .zip file containing all the scan images it took more than 40 min, way longer than the typical time needed by an expert to manually scan the flotation chambers of the FLOTAC with an optical microscope.

However, after several hardware and software fixes, currently the whole process, for both chambers, takes <15 min, in line with the time required when done manually.

Apart from the time performances, the KFM system shows great robustness to adverse conditions, given the fact that the system is closed inside a steel and plastic shell, and the integrated battery enables its usage without being plugged, with an autonomy of 8 h of continuous scanning.

### 5.2 Competitors submissions performances

The test set adopted for this challenge changed during the competition period. Indeed, initially, a dataset available for the challenge until two days before the end is provided, but then a bigger one is used to test the generalization ability of the methodologies proposed by the teams. Table 2 reports the results of systems provided by the participants. For the sake of completeness, we show the performance of the Saksham_Aggarwal team, even if the members did not complete the challenge, making their participation not valid. When the first test set is used, the PARASITE solution exceeds the MIVIA Lab solution by a margin of ~0.06. This trend did not change when a different set of data is considered for the evaluation, since the first solution outperforms the second one by ~0.09. Interestingly, as expected, the two models show signs of overfitting, since the F1-Scores for both solutions dropped when used to make predictions on the second test set. However, given the large difference between the first and the second test set in terms of size, with the second bigger than the first one, a drop in performances was expected.

**Table 2:** AI-KFM 2022 Challenge F1-Scores and execution times (in terms of images per second).

**Team**	**Test 1**	**Test 2**	**Time**
PARASITE	0.7923	0.6275	2.5
Saksham_Aggarwal	0.7736	–	–
MIVIA Lab	0.7330	0.5137	–

The PARASITE solution achieves an inference rate of 2.5 images per second, processing images at a resolution of 1,600 × 1,200 pixels. This performance was measured on a workstation equipped with an NVIDIA RTX 3090 GPU, an Intel(R) Core(TM) i7-10700KF CPU, and 32 GB of DDR4 RAM. The approach utilizes a Mask R-CNN model comprising × 64 million parameters.

**Figure 14:**
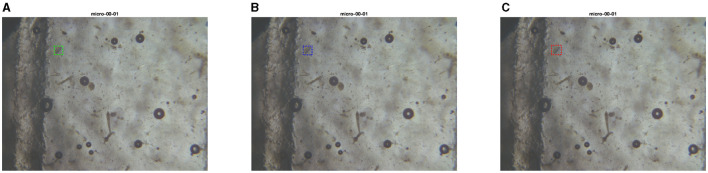
Test set image where models from PARASITE and MIVIA Lab have similar performances. The first image is the ground truth **(A)**, the second one is the inference with the PARASITE solution **(B)**, the third one is the inference with the MIVIA Lab solution **(C)**.

**Figure 15:**
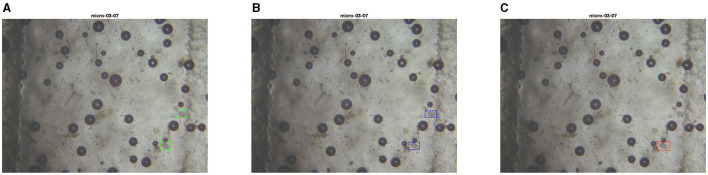
Test set image where PARASITE model makes an extra correct prediction. The first image is the ground truth **(A)**, the second one is the inference with the PARASITE solution **(B)**, the third one is the inference with the MIVIA Lab solution **(C)**.

**Figure 16:**
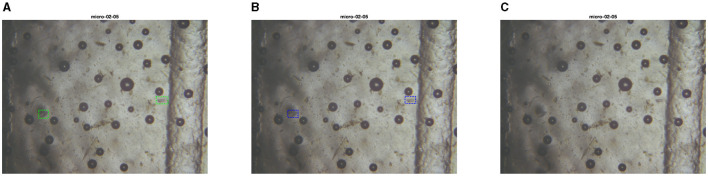
Test set image where only the PARASITE model is capable of recognizing parasite eggs. The first image is the ground truth **(A)**, the second one is the inference with the PARASITE solution **(B)**, the third one is the inference with the MIVIA Lab solution **(C)**.

**Figure 17:**
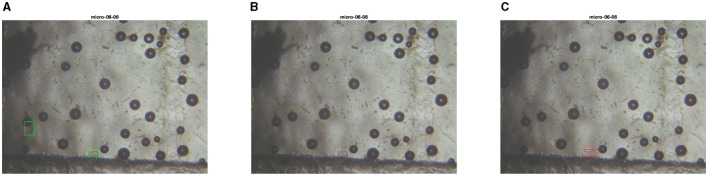
Test set image where only the MIVIA Lab model is capable of recognizing at least a parasite egg. The first image is the ground truth **(A)**, the second one is the inference with the PARASITE solution **(B)**, the third one is the inference with the MIVIA Lab solution **(C)**.

Although the MIVIA Lab team did not participate in Track 2, their submitted solution incorporated a U-Net model with an input size of 572 × 572 pixels and × 28 million parameters. Predictions results on some images from the test set can be seen in Figures 14–17.

## 6 Discussion

The hardware provided by the KFM system is an inexpensive (~600€) but efficient solution which can be brought and used directly in livestock farmings, both by researchers and by inexpert operators, given its ease to use. This component is then enhanced thanks to an automatic detection system, which core will be based on the results obtained in the first AI-KFM Challenge, which can be intended to serve as a standardized common experimental protocol in the context of automatic detection, classification and count of parasites eggs. This challenge provided researchers with more than 2,500 images acquired by using a KFM equipped with FLOTAC and Mini-FLOTAC devices. Three teams took part to the challenge, but only two performed all the stages required to be part of the final score, therefore these two systems will be sources of inspiration for the KFM final model.

These two solutions are not so far from already existing approaches considered in the current literature as state-of-the-art for parasite egg segmentation. For instance, Mask R-CNN (as the one used by the PARASITE team) is an architecture already adopted for the detection and segmentation of an egg of a specific parasite (*Caenorhabditis elegans* nematode) (Fudickar et al., 2021). This solution has been proven to be reliable since it reaches a value of 0.958 in F1-Score. Similarly, U-Net is one of the most common architectures adopted for biomedical segmentation and, in particular, for parasites segmentation, as reported in several papers (Górriz et al., 2018; Mirzaei et al., 2022).

What makes the solutions in this paper different from those reported in the literature is the processing pipeline, intended to process images to locate the parasite eggs. Indeed, the two submitted solutions adopt two different data processing approaches able to increase the overall performance. In particular, in the first solution, thanks to the air bubbles filtering, there is a decrease in false positives and, consequently, an increase in precision and, therefore, in F1-Score. In the second solution, a mathematical morphology on threshold masks obtained after k-means clustering is adopted in order to remove spurious false positives, resulting in a precision increase as well and, therefore, in an F1-Score increase.

Unfortunately, neither the adoption of these networks nor these pre-processing approaches make the system reach F1-Score values comparable to the ones obtained with systems reported in the papers cited before (Bosco et al., 2014; Godber et al., 2015; de Castro et al., 2017; Cools et al., 2019). In any case, given the improvement brought by the pre-processing pipeline, a future solution could involve a different network but the same pipeline.

As noted in the previous section, the results, while promising, indicate that significant work remains to achieve a fully reliable model for the KFM system. Importantly, the provided dataset closely mirrors real-world conditions, capturing the actual environment that the KFM's camera will encounter, including dirt and focus imperfections. This realistic representation enhances the robustness of the proposed models compared to state-of-the-art solutions, which typically rely on clean datasets that depict idealized and less plausible sample conditions.

As the PARASITE won the first edition of the AI-KFM challenge, the team had the opportunity to show their solution during the International Conference on Image Analysis and Processing (ICIAP) 2021 conference. Thanks to these contributions, it is clear that post-processing steps are crucial in order to obtain better and more reliable detection results. Also, despite the small number of submissions received in this first edition, we are satisfied by the overall experience matured organizing and leading the competition. All the data and results, as well as the current leaderboard, will be kept online, to support researchers and practitioners in the field.

## 7 Conclusions

In this paper, we detailed the materials and methods used to establish the first AI-KFM Challenge, beginning with the apparatuses from the FLOTAC family and the Kubic FLOTAC Microscope. These tools enabled us to automatically generate a dataset of samples from real on-field acquisitions.

Utilizing these resources, we provided a realistic and extensive dataset for both competitors and researchers interested in training models for parasite egg detection. Existing datasets are either private or do not accurately represent field conditions, as they often contain images with a single, centrally-focused egg. In contrast, our dataset includes images that reflect the variability of autonomous scan processes, where eggs may be occluded, out of focus, or located at the image edges.

Furthermore, we established a baseline as a reference point for researchers developing models for this challenging task.

Building on these initial results, future work will involve further exploration of new architectures for the detection system and the development of advanced pre-processing techniques to enhance sample images.

Given all these considerations, we are already planning the next edition of the AI-KFM challenge, in which participants will compete on images containing different parasites in different hosts, collected by using heterogeneous devices, including commercial ones.

The best submissions from the first and the next editions will be deepened in order to understand what is the best processing and prediction pipeline for KFM automatic parasite eggs detection from samples scans.

Moreover, new datasets with new classes coming from different parasites (Fasciola, Paramphistoma, Strongyloides, etc.) are currently being generated in order to build more capable and useful prediction models, therefore the KFM system will become compatible with more and more use cases.

Finally, one improvement which is planned to include is the capability of the system to detect parasite eggs without an Internet connection, by bringing the CNN inside an optimized version of the KFM hardware, so that the system becomes an embedded AI board which works in any condition and in any place.

## Data Availability

The original contributions presented in the study are included in the article/Supplementary material, further inquiries can be directed to the corresponding author.

## Footnotes

^1^AI-KFM 2022 Challenge: https://sites.google.com/view/ai-kfm2022/.

^2^AI-KFM 2022 Dataset: https://www.kaggle.com/competitions/ai-kfm-2022/data/.

## References

[B1] Abdulla W. (2017). Mask R-CNN for object detection and instance segmentation on keras and tensorflow. Available at: https://github.com/matterport/Mask_RCNN (accessed July 19, 2023).

[B2] AlDahoul N. Karim H. A. Momo M. A. Escobar F. I. F. Magallanes V. A. Tan M. J. T. (2023). Parasitic egg recognition using convolution and attention network. Sci. Rep. 13:14475. 10.1038/s41598-023-41711-3 37660120 PMC10475085

[B3] Barda B. D. Rinaldi L. Ianniello D. Zepherine H. Salvo F. Sadutshang T. . (2013). Mini-flotac, an innovative direct diagnostic technique for intestinal parasitic infections: experience from the field. PLoS Negl. Trop. Dis. 7:e2344. 10.1371/journal.pntd.0002344 23936577 PMC3731229

[B4] Bosco A. Maurelli M. P. Ianniello D. Morgoglione M. E. Amadesi A. Coles G. C. . (2018). The recovery of added nematode eggs from horse and sheep faeces by three methods. BMC Vet. Res. 14, 1–6. 10.1186/s12917-017-1326-7 29304858 PMC5756441

[B5] Bosco A. Rinaldi L. Maurelli M. Musella V. Coles G. Cringoli G. . (2014). The comparison of flotac, fecpak and mcmaster techniques for nematode egg counts in cattle. Acta Parasitol. 59, 625–628. 10.2478/s11686-014-0282-7 25236271

[B6] Cain J. L. Slusarewicz P. Rutledge M. H. McVey M. R. Wielgus K. M. Zynda H. M. . (2020). Diagnostic performance of mcmaster, wisconsin, and automated egg counting techniques for enumeration of equine strongyle eggs in fecal samples. Vet. Parasitol. 284:109199. 10.1016/j.vetpar.2020.109199 32801106

[B7] Charlier J. Rinaldi L. Musella V. Ploeger H. W. Chartier C. Vineer H. R. . (2020). Initial assessment of the economic burden of major parasitic helminth infections to the ruminant livestock industry in Europe. Prev. Vet. Med. 182:105103. 10.1016/j.prevetmed.2020.105103 32750638

[B8] Cools P. Vlaminck J. Albonico M. Ame S. Ayana M. Cringoli G. . (2019). Diagnostic performance of qpcr, kato-katz thick smear, mini-flotac and fecpakg2 for the detection and quantification of soil-transmitted helminths in three endemic countries. PLoS Negl. Trop. Dis. 13:e0007446. 10.1371/journal.pntd.0007446 31369558 PMC6675048

[B9] Cringoli G. Amadesi A. Maurelli M. P. Celano B. Piantadosi G. Bosco A. . (2021). The kubic flotac microscope (KFM): a new compact digital microscope for helminth egg counts. Parasitology 148, 427–434. 10.1017/S003118202000219X 33213534 PMC7938342

[B10] Cringoli G. Maurelli M. P. Levecke B. Bosco A. Vercruysse J. Utzinger J. . (2017). The mini-flotac technique for the diagnosis of helminth and protozoan infections in humans and animals. Nat. Protoc. 12, 1723–1732. 10.1038/nprot.2017.067 28771238

[B11] Cringoli G. Rinaldi L. Maurelli M. P. Utzinger J. (2010). Flotac: new multivalent techniques for qualitative and quantitative copromicroscopic diagnosis of parasites in animals and humans. Nat. Protoc. 5, 503–515. 10.1038/nprot.2009.235 20203667

[B12] Dai J. He K. Sun J. (2015). “Boxsup: exploiting bounding boxes to supervise convolutional networks for semantic segmentation,” in Proceedings of the IEEE International Conference on Computer Vision (Santiago: IEEE), 1635–1643. 10.1109/ICCV.2015.191

[B13] de Castro L. L. D. Abrahão C. L. Buzatti A. Molento M. B. Bastianetto E. Rodrigues D. S. . (2017). Comparison of mcmaster and mini-flotac fecal egg counting techniques in cattle and horses. Vet. Parasitol. Reg. Stud. Rep. 10, 132–135. 10.1016/j.vprsr.2017.10.003 31014585

[B14] Dougherty E. (2018). Mathematical Morphology in Image Processing, Volume 1. Boca Raton, FL: CRC Press. 10.1201/9781482277234

[B15] Elghryani N. Crispell J. Ebrahimi R. Krivoruchko M. Lobaskin V. McOwan T. . (2020). Preliminary evaluation of a novel, fully automated, telenostic device for rapid field-diagnosis of cattle parasites. Parasitology 147, 1249–1253. 10.1017/S0031182020001031 32576299 PMC10317713

[B16] Fudickar S. Nustede E. J. Dreyer E. Bornhorst J. (2021). Mask r-cnn based c. elegans detection with a diy microscope. Biosensors 11:257. 10.3390/bios11080257 34436059 PMC8391161

[B17] Godber O. F. Phythian C. J. Bosco A. Ianniello D. Coles G. Rinaldi L. . (2015). A comparison of the fecpak and mini-flotac faecal egg counting techniques. Vet. Parasitol. 207, 342–345. 10.1016/j.vetpar.2014.12.029 25579397

[B18] Górriz M. Aparicio A. Raventós B. Vilaplana V. Sayrol E. López-Codina D. (2018). “Leishmaniasis parasite segmentation and classification using deep learning,” in Articulated Motion and Deformable Objects, eds. F. J. Perales, and J. Kittler (Cham: Springer International Publishing), 53–62. 10.1007/978-3-319-94544-6_6

[B19] Gröger M. Borisov V. Kasneci G. (2022). “Boxshrink: from bounding boxes to segmentation masks,” in Workshop on Medical Image Learning with Limited and Noisy Data (Cham: Springer), 65–75. 10.1007/978-3-031-16760-7_7

[B20] He K. Gkioxari G. Dollár P. Girshick R. (2017). “Mask R-CNN,” in 2017 IEEE International Conference on Computer Vision (ICCV) (Venice: IEEE), 2980–2988. 10.1109/ICCV.2017.322

[B21] He K. Zhang X. Ren S. Sun J. (2016). “Deep residual learning for image recognition,” in Proceedings of the IEEE Conference on Computer Vision and Pattern Recognition (Las Vegas, NV: IEEE), 770–778. 10.1109/CVPR.2016.90

[B22] Hicks S. A. Strümke I. Thambawita V. Hammou M. Riegler M. A. Halvorsen P. . (2022). On evaluation metrics for medical applications of artificial intelligence. Sci. Rep. 12:5979. 10.1038/s41598-022-09954-8 35395867 PMC8993826

[B23] Inácio S. V. Ferreira Gomes J. Xavier Falcão A. Nagase Suzuki C. T. Bertequini Nagata W. Nery Loiola S. H. . (2020). Automated diagnosis of canine gastrointestinal parasites using image analysis. Pathogens 9:139. 10.3390/pathogens9020139 32093178 PMC7169455

[B24] Inácio S. V. Gomes J. F. Falcão A. X. Martins dos Santos B. Soares F. A. Nery Loiola S. H. . (2021). Automated diagnostics: advances in the diagnosis of intestinal parasitic infections in humans and animals. Front. Vet. Sci. 8:715406. 10.3389/fvets.2021.715406 34888371 PMC8650151

[B25] Kervadec H. Bouchtiba J. Desrosiers C. Granger E. Dolz J. Ayed I. B. . (2019). “Boundary loss for highly unbalanced segmentation,” in International Conference on Medical Imaging with Deep Learning (PMLR), 285–296.

[B26] Krizhevsky A. Sutskever I. Hinton G. E. (2012). ImageNet classification with deep convolutional neural networks. Commun. ACM 60, 84–90.

[B27] Kumar S. Arif T. Ahamad G. Chaudhary A. A. Khan S. Ali M. A. . (2023a). An efficient and effective framework for intestinal parasite egg detection using yolov5. Diagnostics 13:2978. 10.3390/diagnostics13182978 37761346 PMC10527934

[B28] Kumar S. Arif T. Alotaibi A. S. Malik M. B. Manhas J. (2023b). Advances towards automatic detection and classification of parasites microscopic images using deep convolutional neural network: methods, models and research directions. Arch. Comput. Methods Eng. 30, 2013–2039. 10.1007/s11831-022-09858-w 36531561 PMC9734923

[B29] Liu L. Cheng J. Quan Q. Wu F.-X. Wang Y.-P. Wang J. . (2020). A survey on u-shaped networks in medical image segmentations. Neurocomputing 409, 244–258. 10.1016/j.neucom.2020.05.07038310297

[B30] Lu Q. Liu G. Xiao C. Hu C. Zhang S. Xu R. X. . (2018). A modular, open-source, slide-scanning microscope for diagnostic applications in resource-constrained settings. PLoS ONE 13:e0194063. 10.1371/journal.pone.0194063 29543835 PMC5854341

[B31] Maurizio A. Perrucci S. Tamponi C. Scala A. Cassini R. Rinaldi L. . (2023). Control of gastrointestinal helminths in small ruminants to prevent anthelmintic resistance: the italian experience. Parasitology 150, 1105–1118. 10.1017/S0031182023000343 37039466 PMC10801368

[B32] Mayo P. Anantrasirichai N. Chalidabhongse T. H. Palasuwan D. Achim A. (2022). Detection of parasitic eggs from microscopy images and the emergence of a new dataset. arXiv [Preprint]. arXiv:2203.02940. 10.48550/arXiv.2203.02940

[B33] Mirzaei O. Güler E. Akkaya N. Bilgehan B. Süer K. (2022). Automated early-stage enterobius vermicularis diagnosis using segmentation model applied to the deep learning method. 10.21203/rs.3.rs-2171052/v1

[B34] Nagamori Y. Hall Sedlak R. DeRosa A. Pullins A. Cree T. Loenser M. . (2020). Evaluation of the vetscan imagyst: an in-clinic canine and feline fecal parasite detection system integrated with a deep learning algorithm. Parasit. Vectors 13, 1–10. 10.1186/s13071-020-04215-x 32653042 PMC7353785

[B35] Nagamori Y. Sedlak R. H. DeRosa A. Pullins A. Cree T. Loenser M. . (2021). Further evaluation and validation of the vetscan imagyst: in-clinic feline and canine fecal parasite detection system integrated with a deep learning algorithm. Parasit. Vectors 14, 1–12. 10.1186/s13071-021-04591-y 33514412 PMC7844936

[B36] Naing K. M. Boonsang S. Chuwongin S. Kittichai V. Tongloy T. Prommongkol S. . (2022). Automatic recognition of parasitic products in stool examination using object detection approach. PeerJ Comput. Sci. 8:e1065. 10.7717/peerj-cs.1065 36092001 PMC9455271

[B37] Palasuwan D. Naruenatthanaset K. Kobchaisawat T. Chalidabhongse T. H. Nunthanasup N. Boonpeng K. . (2022). “Parasitic egg detection and classification in microscopic images,” in IEEE Dataport.

[B38] Pedraza A. Ruiz-Santaquiteria J. Deniz O. Bueno G. (2022). “Parasitic egg detection and classification with transformer-based architectures,” in 2022 IEEE International Conference on Image Processing (ICIP) (Bordeaux: IEEE), 4301–4305. 10.1109/ICIP46576.2022.9897846

[B39] Peña-Espinoza M. (2018). Drug resistance in parasitic helminths of veterinary importance in chile: status review and research needs. Austral J. Vet. Sci. 50, 65–76. 10.4067/S0719-8132201800020006527315006

[B40] Rainio O. Teuho J. Klén R. (2024). Evaluation metrics and statistical tests for machine learning. Sci. Rep. 14:6086. 10.1038/s41598-024-56706-x 38480847 PMC10937649

[B41] Rajasekar S. J. S. Jaswal G. Perumal V. Ravi S. Dutt V. (2023). “Parasite. AI-an automated parasitic egg detection model from microscopic images of fecal smears using deep learning techniques,” in 2023 International Conference on Advances in Computing, Communication and Applied Informatics (ACCAI) (IEEE), 1–9.

[B42] Rajchl M. Lee M. C. Oktay O. Kamnitsas K. Passerat-Palmbach J. Bai W. . (2016). Deepcut: object segmentation from bounding box annotations using convolutional neural networks. IEEE Trans. Med. Imaging 36, 674–683. 10.1109/TMI.2016.2621185 27845654 PMC7115996

[B43] Redmon J. Divvala S. Girshick R. Farhadi A. (2016). “You only look once: unified, real-time object detection,” in Proceedings of the IEEE Conference on Computer Vision and Pattern Recognition (Las Vegas, NV: IEEE), 779–788. 10.1109/CVPR.2016.91

[B44] Ren S. He K. Girshick R. Sun J. (2017). Faster R-CNN: Towards real-time object detection with region proposal networks. IEEE Trans. Pattern Ana. Mach. Intell. 39, 1137–1149. 10.1109/TPAMI.2016.2577031 27295650

[B45] Ruiz-Santaquiteria J. Pedraza A. Vallez N. Velasco A. (2022). “Parasitic egg detection with a deep learning ensemble,” in 2022 IEEE International Conference on Image Processing (ICIP) (Bordeaux: IEEE), 4283–4286. 10.1109/ICIP46576.2022.9897858

[B46] Sabatini G. A. de Almeida Borges F. Claerebout E. Gianechini L. S. Höglund J. Kaplan R. M. . (2023). Practical guide to the diagnostics of ruminant gastrointestinal nematodes, liver fluke and lungworm infection: interpretation and usability of results. Parasit. Vectors 16:58. 10.1186/s13071-023-05680-w 36755300 PMC9906602

[B47] Salvi M. Acharya U. R. Molinari F. Meiburger K. M. (2021). The impact of pre-and post-image processing techniques on deep learning frameworks: a comprehensive review for digital pathology image analysis. Comput. Biol. Med. 128:104129. 10.1016/j.compbiomed.2020.104129 33254082

[B48] Scare J. Slusarewicz P. Noel M. Wielgus K. Nielsen M. (2017). Evaluation of accuracy and precision of a smartphone based automated parasite egg counting system in comparison to the mcmaster and mini-flotac methods. Vet. Parasitol. 247, 85–92. 10.1016/j.vetpar.2017.10.005 29080771

[B49] Slusarewicz P. Pagano S. Mills C. Popa G. Chow K. M. Mendenhall M. . (2016). Automated parasite faecal egg counting using fluorescence labelling, smartphone image capture and computational image analysis. Int. J. Parasitol. 46, 485–493. 10.1016/j.ijpara.2016.02.004 27025771

[B50] Sukas S. Van Dorst B. Kryj A. Lagatie O. De Malsche W. Stuyver L. J. (2019). Development of a lab-on-a-disk platform with digital imaging for identification and counting of parasite eggs in human and animal stool. Micromachines 10:852. 10.3390/mi10120852 31817458 PMC6952989

[B51] Suwannaphong T. Chavana S. Tongsom S. Palasuwan D. Chalidabhongse T. H. Anantrasirichai N. . (2021). Parasitic egg detection and classification in low-cost microscopic images using transfer learning. arXiv [Preprint]. arXiv:2107.00968. 10.48550/arXiv.2107:00968

[B52] Suwannaphong T. Chavana S. Tongsom S. Palasuwan D. Chalidabhongse T. H. Anantrasirichai N. . (2023). Parasitic egg detection and classification in low-cost microscopic images using transfer learning. SN Comput. Sci. 5:82. 10.1007/s42979-023-02406-8

[B53] Tyson F. Dalesman S. Brophy P. M. Morphew R. M. (2020). Novel equine faecal egg diagnostics: validation of the fecpakg2. Animals 10:1254. 10.3390/ani10081254 32717982 PMC7459939

[B54] Vande Velde F. Charlier J. Claerebout E. (2018). Farmer behavior and gastrointestinal nematodes in ruminant livestock-uptake of sustainable control approaches. Front. Vet. Sci. 5:255. 10.3389/fvets.2018.00255 30386785 PMC6198092

[B55] Wang Y. He Z. Huang S. Du H. (2022). “A robust ensemble model for parasitic egg detection and classification,” in 2022 IEEE International Conference on Image Processing (ICIP) (Bordeaux: IEEE), 4258–4262. 10.1109/ICIP46576.2022.9897192

[B56] Wright L. (2019). Ranger - a synergistic optimizer. Available at: https://github.com/lessw2020/Ranger-Deep-Learning-Optimizer (accessed June 14, 2019).

[B57] Zaitoun N. M. Aqel M. J. (2015). Survey on image segmentation techniques. Procedia Comput. Sci. 65, 797–806. 10.1016/j.procs.2015.09.027

